# Comparative genomic analysis uncovered phylogenetic diversity, evolution of virulence factors, and horizontal gene transfer events in tomato bacterial spot *Xanthomonas euvesicatoria*

**DOI:** 10.3389/fmicb.2024.1487917

**Published:** 2024-11-05

**Authors:** Chien-Jui Huang, Ting-Li Wu, Yu-Lin Wu, Ruei-Shiuan Wang, Yao-Cheng Lin

**Affiliations:** ^1^Department of Plant Medicine, National Chiayi University, Chiayi, Taiwan; ^2^Biotechnology Center in Southern Taiwan, Academia Sinica, Tainan, Taiwan; ^3^Agriltural Biotechnology Research Center, Academia Sinica, Taipei, Taiwan

**Keywords:** *Xanthomonas euvesicatoria*, bacterial spot, microbial evolution, comparative genomics, virulence factors, microbial diversity

## Abstract

**Introduction:**

Bacterial spot, caused by diverse xanthomonads classified into four lineages within three species, poses a significant threat to global pepper and tomato production. In Taiwan, tomato bacterial spot xanthomonads phylogenetically related to an atypical *Xanthomonas euvesicatoria* pv. *perforans* (*Xep*) strain NI1 from Nigeria were found.

**Methods:**

To investigate the genetic structure of Taiwanese *Xep* strains and determine the phylogenetic position of the atypical strains, we completed high-quality, gap-free, circularized genomes of seven Taiwanese *Xep* strains and performed comparative genomic analyses with genomes of *X. euvesicatoria* pathovars. Average nucleotide identity, core genome analysis, and phylogenomic analysis were conducted.

**Results:**

Three sequenced strains were identified as typical *Xep*, while four clustered with the atypical strain NI1, forming a distinct genomovar within X. euvesicatoria, proposed as *X. euvesicatoria* genomovar taiwanensis (*Xet*). This new lineage likely originated in Taiwan and spread to Nigeria through global seed trade. At the genomovar level, chromosomes remained conserved among Taiwanese strains, while plasmids likely contributed to bacterial virulence, avirulence, and field fitness. Gap-free genomes revealed associations between the evolution of type III effectors, horizontal gene transfer events, plasmid diversity, and recombination.

**Discussion:**

This study highlights the critical roles of horizontal gene transfer and plasmids in shaping the genetic makeup, evolution, and environmental adaptation of plant pathogenic xanthomonads. The identification of a new genomovar, *X. euvesicatoria* genomovar *taiwanensis*, provides insights into the diversity and global spread of bacterial spot pathogens through seed trade.

## Introduction

Bacterial spot disease, caused by various species of the genus Xanthomonas, poses a significant threat to global tomato (*Solanum lycopersicum*) and pepper (*Capsicum* spp.) production, leading to considerable economic losses (Osdaghi et al., 2021). Although precise estimates of economic losses are scarce, it is estimated that annual damages caused by *Xanthomonas* in tomato can range from $3,000 to $7,000 USD per hectare in Florida and could exceed $7.8 million USD during an outbreak in Northwest Ohio and Southeast Michigan alone.^1^ Yield reductions of up to 66% under favorable conditions have been reported in United States (Pohronezny and Volin, 1983). In Taiwan, bacterial spot is a prevalent issue in tomato cultivation, affecting crop yield and quality (Leu et al., 2010).

The genus *Xanthomonas* spp. represents a large group of plant pathogenic gram-negative bacteria, with more than 400 plant hosts recorded as susceptible to xanthomonads (Timilsina et al., 2020). In particular, bacterial spot poses a significant threat to global pepper and tomato production (Osdaghi et al., 2021). The complexity of this disease from the perspective of the pathogen is considerable. Initially, causal xanthomonads were thought to consist of two genetically distinct clonal groups; however, later studies identified four distinct groups (Jones et al., 2004). Currently, bacterial spot-causing *Xanthomonas* spp. are classified into four distinct lineages within three species: *Xanthomonas euvesicatoria* pv. *euvesicatoria* (*Xee*), *X. euvesicatoria* pv. *perforans* (*Xep*), *X. hortorum* pv. *gardneri*, and *X. vesicatoria*, based on phylogenomic analysis of whole genome sequences (Jones et al., 2004; Constantin et al., 2016; Osdaghi et al., 2021).

Among these lineages, *Xep* is particularly diverse, with new phylogenetic groups being continuously identified (Abrahamian et al., 2021). For example, *Xep* strains collected in Florida during the 1990s were initially classified into a single phylogenetic group (Schwartz et al., 2015; Timilsina et al., 2019), but six distinct phylogenetic groups were recognized among Florida strains by 2017 (Newberry et al., 2019; Abrahamian et al., 2021). Australian *Xep* strains form unique phylogenetic clusters that are distinct from those found in other regions (Roach et al., 2019). Beyond these well-characterized lineages, atypical *Xep* strains causing tomato bacterial spot have been identified in Nigeria since 2012 (Jibrin et al., 2015; Timilsina et al., 2015; Jibrin et al., 2018; Jibrin et al., 2022). Both atypical and typical *Xep* strains are amylolytic, and no differences in phenotypic characteristics between atypical and typical *Xep* strains have been reported (Jibrin et al., 2015). Genetically, these atypical strains can be distinguished by multilocus sequence analysis (MLSA) and phylogenetic analysis of *gapA* sequences (Timilsina et al., 2015; Timilsina et al., 2016). Moreover, the core genome phylogeny and pangenomic analysis suggest the atypical Nigerian strain NI1 was intermediate between *Xee* and typical *Xep* strains but more closely related to *X. euvesicatoria* pv. *allii* (Jibrin et al., 2018). Comparative genomic analysis further revealed that the atypical Nigerian strain NI1 differed from typical *Xep* strains in the composition of the type four secretion system and lipopolysaccharide biosynthetic gene clusters (Jibrin et al., 2022). MLSA results and draft genome comparisons of Nigerian strain NI1 suggested that these atypical *Xep* strains may have evolved through recombination between *Xee* and *Xep* (Timilsina et al., 2015; Jibrin et al., 2018). Core genome phylogeny indicated that strain NI1, along with six *Xep* strains isolated from peppers in Taiwan in 2019, formed a cluster named TP-2019 (Parajuli et al., 2024). However, no tomato strains of *Xep* have been identified in Taiwan in this cluster (Chen et al., 2024).

In Taiwan, bacterial spot on tomatoes and peppers is predominantly caused by *Xee* and *Xep* (Leu et al., 2010; Constantin et al., 2016). There has been a notable transition in bacterial spot xanthomonad populations in Taiwan. Burlakoti et al. (2018) reported that all pepper strains and 95% of tomato strains collected between 1989 and 1999 were *Xee*, with no *Xep* strains found during this period. Although *Xep* was first reported in tomatoes in Taiwan in 2010 (Leu et al., 2010), 22% of the tomato strains collected by the World Vegetable Center between 2000 and 2009 were identified as *Xep* (Burlakoti et al., 2018). Since 2010, *Xep* has become the dominant causal agent of tomato spot disease in Taiwan, with 99% of the tomato strains identified as *Xep* (Burlakoti et al., 2018).

Despite the prevalence of *Xep*, recent studies have revealed limited genetic diversity among Taiwanese *Xep* strains. Analysis of the draft genome assemblies of 11 *Xep* strains from Taiwan showed no evidence of recombination with other tomato bacterial spot *Xanthomonas* species, indicating a narrow genetic background (Chen et al., 2024). Interestingly, based on the *gapA* phylogeny, we found that several *Xep* strains isolated in Taiwan since 1996 were phylogenetically close to the representative atypical strain NI1. Understanding the genetic diversity and evolutionary relationships of these atypical strains is crucial for accurate pathogen identification, disease management, and elucidation of the mechanisms underlying their pathogenicity and adaptation. However, there is limited information regarding the complete genome composition of *Xep* in Taiwan.

In this study, we aimed to obtain high-quality complete genomes of atypical Xep strains from Taiwan and clarify their phylogenetic position within diverse lineages of *X. euvesicatoria* through comparative genomic analysis. Specifically, we sought to decipher the genetic basis of the so-called *Xep* by analyzing the complete genome sequences of seven strains collected over different years and regions in Taiwan, including four copper-resistant (Cu^R^) strains. The high-quality genome assemblies and annotations of these seven strains were compared with those of other *X. euvesicatoria* genomes through genome analysis to reveal their phylogenetic positions and genetic diversity. Based on our findings, we propose classifying this new lineage as *X. euvesicatoria* genomovar *taiwanensis* (*Xet*). By analyzing completely circularized genome assemblies, we provided detailed insights into plasmid composition, evidence of recombination and horizontal gene transfer, and the evolution of virulence factors in the sequenced genomes.

## Materials and methods

### Collection of strains

Seven originally identified *Xep* strains were selected based on the year and location of isolation (Lai et al., 2021). Two strains, T0709-01 and T0709-03, were isolated in 2016, and four strains, T0319-01, XpT2, XpT32, and XpT39, were isolated in 2018. An *Xep* isolate, XTN47, obtained in 1996 (Leu et al., 2010), was included in this study. Strains XTN47, T0709-01, and T0709-03 were obtained from Chiayi, T0319-01 from Shueishang, XpT2 from Taipao, and XpT32 and XpT39 from Liujiao. All strains were isolated from tomato leaves with symptoms of bacterial spot, and their pathogenicity was confirmed by fulfilling Koch’s postulates (Table 1).

**Table 1:** Genome information of sequenced strains.

Strain	Copper	Phenotype	Size (bp)	GC %	Genes	GenBank	Isolated location	Isolated year
T0319-01^T^	Cu^R^	Atypical	4,974,947	64.9	3,946	CP137539	Shueishang	2018
pT0319-01.1			255,122	59.7	240	CP137540		
pT0319-01.2			114,517	64.3	135	CP137541		
pT0319-01.3			62,580	60.2	52	CP137542		
XpT32	Cu^R^	Atypical	4,946,329	64.9	3,847	CP137536	Liujiao	2018
pXT32.1			251,985	59.6	238	CP137537		
pXT32.2			62,585	60.2	54	CP137538		
XpT39	Cu^R^	Atypical	4,987,245	64.9	4,076	CP137532	Liujiao	2018
pXT39.1			250,793	59.6	233	CP137533		
pXT39.2			62,596	60.2	55	CP137534		
pXT39.3			30,950	59.7	40	CP137535		
T0709-01	Cu^R^	Typical	4,915,631	65.0	4,037	CP157486	Chiayi	2016
p0709-01.1			249,567	59.6	231	CP157487		
p0709-01.2			82,027	60.2	78	CP157488		
p0709-01.3			28,181	63.1	36	CP157489		
T0709-03	Cu^S^	Typical	4,915,609	65.0	4,036	CP157490	Chiayi	2016
pT0709-03.1			82,042	60.2	78	CP157491		
pT0709-03.2			28,181	63.1	36	CP157492		
XpT2	Cu^S^	Typical	4,906,464	65	4,024	CP166450	Taipao	2018
pXpT2.1			93,650	57.1	93	CP166451		
pXpT2.2			82,070	60.2	78	CP166452		
pXpT2.3			10,900	61.6	19	CP166453		
XTN47	Cu^S^	ATypical	5,073,981	64.9	4,187	CP166448	Chiayi	1996
pXTN47.1			97,301	60.7	107	CP166449		

T0319-01^T^ = BCRC 81416 https://catalog.bcrc.firdi.org.tw/BcrcContent?bid=81416&rowid=1.

### Nanopore and Illumina sequencing

Genomic DNA (gDNA) from the seven strains was prepared using the NautiaZ Bacteria/Fungi DNA Mini Kit (Nautia Gene, Taipei, Taiwan). The quality of the gDNA was estimated using a Qubit fluorometer (Qiagen), and the fragment size was measured using a capillary electrophoresis instrument TapeStation (Agilent Technologies). Sheared gDNA was selected using BluePippin with a 0.75% agarose gel cassette (Sage Science) to obtain gDNA fragment sizes ranging from 6 to 20 kb. Nanopore sequencing libraries were prepared using the PCR-free ligation-based sequencing kit (SQK-LSK109) with native barcoding expansion (EXP-NBD104) for sample multiplexing. Nanopore sequencing was performed using an Oxford Nanopore GridION device (R9.4 flow cell FLO-MIN106D). In total, we obtained 161 K–183 K Nanopore reads for each strain, with an average Nanopore read length of 8,875 bp and an L50 of 48,138 bp (Supplementary Table S1). For the Illumina sequencing, genomic DNA libraries were prepared for high-throughput sequencing using the Illumina DNA Prep Kit (Illumina, San Diego, CA, United States). Whole-genome shotgun sequencing was performed using an Illumina NovaSeq 6,000 platform (Illumina, San Diego, CA, United States) to generate 150 bp paired-end reads. Sequencing depth achieved an average of 2.8–3.1 million paired-end reads per genome (Supplementary Table S1).

### Genome assembly and gene annotation

Sequencing read quality was evaluated using FastQC (v.0.11.9) (Andrews et al., 2023). Low-quality reads were subsequently removed using Trimmomatic (v.0.36) (Bolger et al., 2014) for Illumina reads and NanoFilt (v.2.6.0) (De Coster et al., 2018) for Nanopore reads. The base quality of Nanopore reads greater than 1 kb was further improved by the corresponding high-quality Illumina reads using FMLRC (v.1.0.0) (Wang et al., 2018). Each strain was assembled using three different assemblers, Canu (v.1.8) (Koren et al., 2017), Flye (v.2.5) (Kolmogorov et al., 2019), and wtdbg2 (v.2.5) (Ruan and Li, 2020), for their ability to handle repetitive regions. The draft assemblies for each strain were then compared and merged using BLASTN (v.2.10.1) (Camacho et al., 2009) with manual inspection to produce a consensus assembly. The per-base accuracy of the assemblies was improved using Pilon (Wang et al., 2014) with high-quality Illumina reads. On average, the final coverage of the assembled genomes exceeded 200X. Genomic sequences of the seven strains were resolved on a single circular chromosome. All plasmid sequences were fully circularized. Genome assembly quality in terms of completeness and contamination was assessed based on the presence and duplication of marker genes using CheckM (Parks et al., 2015) and BUSCO (v5.5.0, −l xanthomonadales_odb10) (Manni et al., 2021) (Supplementary Table S1).

To facilitate whole-genome comparison, the *dnaA* gene was set as the start of the complete genome assembly. Three conserved DnaA boxes have been identified in the *oriC* region between *dnaA* and *dnaN* (da Silva et al., 2002; Yen et al., 2002; Qian et al., 2005). The terminator of replication for each strain was located at approximately 2.5 Mb of the chromosome sequence. Replichores of the three strains were identified using a GC skew plot.

Genome annotation was performed using the local installation of the NCBI Prokaryotic Genome Annotation Pipeline (PGAP) (Tatusova et al., 2016), followed by manual curation. Specifically, the Docker image of PGAP (v.2019-08-22.build3958) was used for the initial genome annotation. The annotations generated by PGAP were then manually inspected using Artemis (Carver et al., 2012) to verify the correctness of the start and stop codons, among other features. A comparative analysis with published *X. euvesicatoria* genomes was conducted using BLASTP (Camacho et al., 2009) to further refine and validate the annotations.

Despite the publication of the first *Xanthomonas* genome (*X. citri* 306) (da Silva et al., 2002) in 2002, followed by numerous subsequent genome sequences and studies, a significant portion of the genome remains functionally uncharacterized. To enhance functional annotation, we used a combined approach with UniProt (Coudert et al., 2023), Blast2GO (Gotz et al., 2008), and Intrproscan (Jones et al., 2014). However, this combined strategy resulted in approximately 20% of the protein-coding genes remaining classified as hypothetical, indicating an unknown function.

### Whole genome comparison and gene family analysis

Two complementary approaches based on the diversity of nucleotide sequences and protein-coding genes have been used for genome-wide analyses to understand overall genome variations. The assembled chromosome and plasmid sequences were compared with published 132 *Xanthomonas* genomes (Supplementary Table S2) to identify conserved and novel sequence elements (Barak et al., 2016; Constantin et al., 2016; Bansal et al., 2018; Fan et al., 2022). The whole-genome similarity metrics of all strains were estimated using the alignment-free approximate sequence mapping algorithm FastANI (v.1.20) (Jain et al., 2018), with default settings. To confirm the taxonomic nomenclature and classification of these strains, genome-based taxonomy was performed using the GGDC database (Meier-Kolthoff et al., 2022).

To further understand the evolution of protein-coding genes in the *Xanthomonas* genomes, we analyzed genes from 139 *Xanthomonas* strains (Supplementary Table S2) to infer orthologous relationships using the Markov Cluster Algorithm (MCL) (Enright et al., 2002). Briefly, genes were compared using all-against-all BLASTP (e-value 10^−5^) (Camacho et al., 2009), and the results were analyzed using the scalable unsupervised cluster algorithm MCL (inflation value 5). Orthologous and paralogous relationships are presented as networks based on e-values. MCL clustering resulted in 8,580 gene families, which were presented as a gene number matrix of 139 genomes. Gene families present in more than one genome were considered the ′pan-genome′ whereas those shared in at least two genomes were considered the ′core-genome′ (Méric et al., 2014).

A total of 1,346 orthologous genes with a strict one-to-one relationship as the ′single copy core gene′ in 139 *Xanthomonas* strains were used to construct the phylogenetic tree. Multiple sequence alignments of the protein sequences in these single-copy core genes were individually performed using MUSCLE (Edgar, 2004), and gaps in the alignments were removed using trimAl (Capella-Gutiérrez et al., 2009). Individual sequence alignments were concatenated to create a single protein sequence for each strain. Maximum likelihood phylogenetic trees were inferred using RAxML-NG (Kozlov et al., 2019) with 1,000 bootstrap replicates (v.1.0.1, −model LG + G8 + F –seed 2 –bs-trees 1,000) and visualized using FigTree.^2^ Genome collinearity analysis was performed using i-ADHoRe 3.0 (Proost et al., 2012) to infer the syntenic regions.

#### Analysis of homologous recombination

We analyzed homologous recombination among *X. euvesicatoria* strains using ClonalFrameML (v1.13; Didelot and Wilson, 2015). The phylogenetic tree used as the starting topology was constructed based on single-copy core genes. Protein alignments were back-translated to coding DNA sequence (CDS) alignments, and phylogeny was inferred using RAxML-NG (Kozlov et al., 2019) with 1,000 bootstrap replicates (v.1.2.0, −model GTR + G –seed 2 –bs-trees 1,000). Pangenome alignment was performed using Cactus (v2.7.0; Hickey et al., 2024). Transition/transversion ratios were determined using Cactus alignment. Recombination parameters were calculated in ClonalFrameML using the hidden Markov model (HMM) with 100 simulations (emsim = 100) to enhance the reliability of the analysis. Additionally, we calculated the impact of introduced recombination events relative to single-point mutations (*r/m*) for each branch in ClonalFrameML. Analyses were conducted using different combinations of strains, as shown in Table 2.

**Table 2:** Rate of recombination between *X. euvesicatoria* lineages.

*Xanthomonas* population	R/θ	δ (SD)	ν	R/m	Event count	Ts/Tv	Event occured (in Node/across distinct strains)
Atypical *Xep* only from Taiwan	0.440	13,997 (947.9)	0.0171	105.58	118	2.15	12/106
Atypical *Xep* only from Taiwan +NI1	0.469	11,144 (751.4)	0.0169	88.45	153	2.16	29/124
Other *Xep*	1.568	8,995 (560.2)	0.0168	236.80	222	2.15	44/178
All atypical *Xep* + other *Xep*	0.777	2,376 (69.0)	0.0136	25.16	848	2.22	632/216
All strains in selected lineage^+^	0.371	80 (0.6)	0.0687	2.04	4,629	2.27	2,535/2,094

A. + Selected lineages correspond to the asterisk (*) highlighted in Figure 3. B. Definitions of parameters. R/θ (Rate of recombination over mutation rate): The rate of recombination divided by the rate of mutation. δ (Delta): Average size of the recombined fragments measured in base pairs. SD: standard deviation. ν (Nu): Probability of recombination per site. R/m (Effect of recombination relative to mutation): The ratio of per-base-pair substitutions introduced by recombination to those introduced by mutation. Tr/Tv (Transition/Transversion ratio): The ratio of transitions to transversions used as an input parameter in the analysis.

#### Phenotypic characterization

The biochemical characteristics of the strains were determined using a Biolog GEM III MicroPlate system (Biolog, Hayward, CA, United States). The amylolytic activity of the strains was tested on nutrient agar (NA; Difco) plates containing 1.5% soluble starch, as described by Stall et al. (1994).

A copper sensitivity test was performed according to the method described by Lai et al. (2021). The three strains were cultured overnight on NA plates and streaked on NA plates supplemented with 0, 0.1, 0.2, 0.4, 0.6, 0.7, 0.8, 1.6, and 3.2 mM CuSO_4_. Stains sensitive, tolerant, or resistant to copper were differentiated by their ability to grow on NA plates with maximum concentrations of ≤0.6, 0.6–0.8 and ≥ 0.8 mM CuSO_4_, respectively, as rated by Behlau et al. (2013) and Marin et al. (2019).

#### Horizontal transfer of copper resistance genes

The horizontal transfer of Cu^R^ genes between different xanthomonad strains was tested according to the method described by Behlau et al. (2012). Strain T0319-01, with copper resistance and rifampicin sensitivity, was used as the donor. Spontaneous rifampicin-resistant mutants of the Cu^S^ strain XTN47Rif were used as recipients. The bacterial strains were mated on NA plates at 28°C for 24 h. After mating, the bacterial cells were scraped, suspended, and plated at dilutions on NA supplemented with rifampicin (50 mg/L) to estimate the recipient population. To select transconjugants, bacterial suspensions were plated at dilutions on NA supplemented with rifampicin (50 mg/L) and 0.8 mM CuSO_4_. The conjugation frequency was calculated as the ratio of the number of transconjugants to the recipient population (Behlau et al., 2012).

## Results

### Genome sequence of seven tomato bacterial spot xanthomonad strains

To investigate the evolutionary divergence and better understand the genomic basis of Taiwanese *X. euvesicatoria* pv. *perforans* (*Xep*) strains, we performed whole-genome sequencing of seven *Xep* strains collected from different locations during 1996–2018 (Table 1). Three typical *Xep* strains (T0709-01, T0709-03, and XpT2) and four atypical *Xep* strains (XpT32, XpT39, T0309-01, and XTN47 (Leu et al., 2010)) were selected. Among these, one typical strain and three atypical strains were characterized as Cu^R^ strains, capable of growing on NA plates supplemented with 0.8 mM CuSO (Lai et al., 2021). Conversely, two other typical *Xep* strains, T0709-03 and XpT2, and an atypical strain isolated in 1996, XTN47, were sensitive to copper.

**Figure 1:**
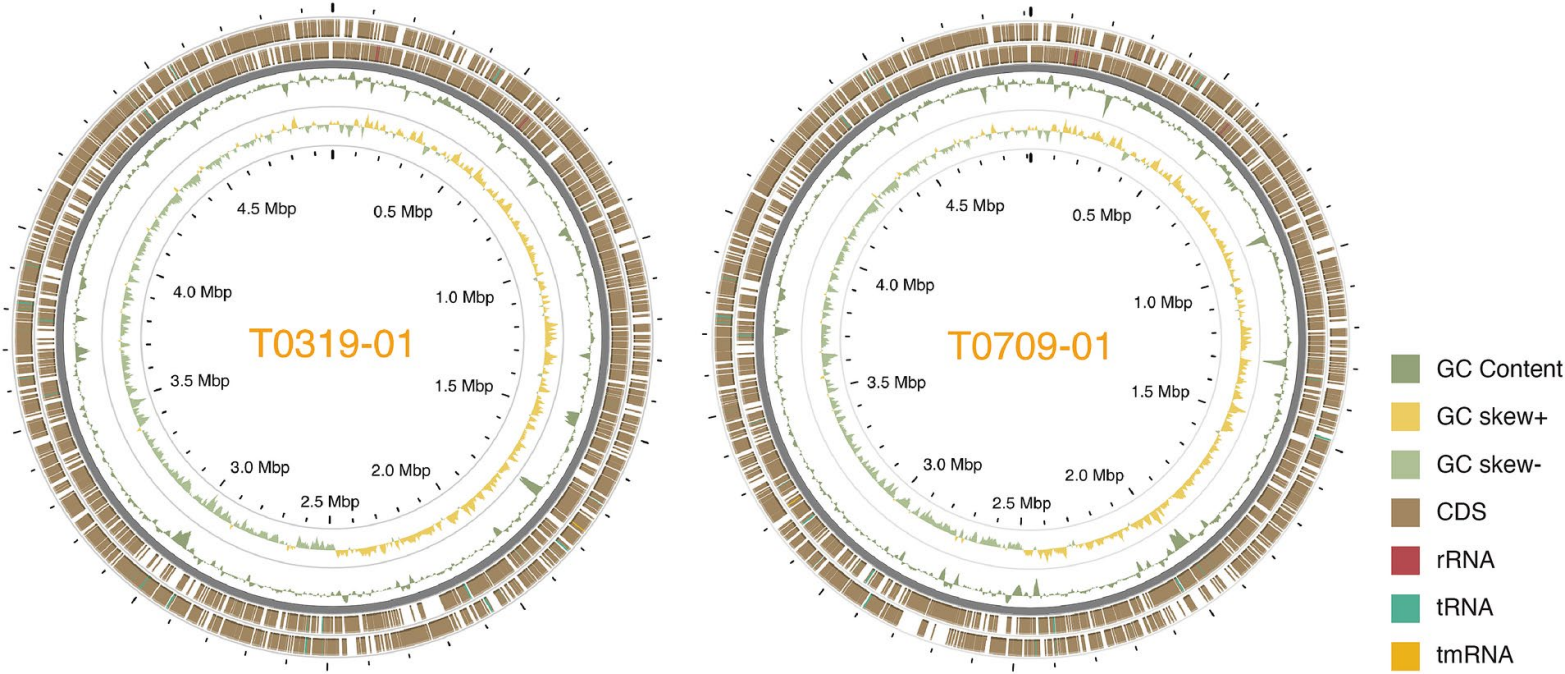
Circos plot of the two Taiwanese *Xep* strains. The outermost two layers represent coding sequences (CDS) on both strands. The next layer shows the GC content, followed by the GC skew of the fourth layer. The innermost layer represented the grid. The GC content and skew were calculated using a window size of 10 kb and a step size of 100 bp.

We used a hybrid assembly approach that combined short-read (Illumina) and long-read (Oxford Nanopore) sequencing technologies in an integrated bioinformatics workflow (Huang et al., 2021). This method allowed us to complete the genomes of all seven strains into gap-free and circularized chromosomes and plasmid sequences (Figure 1). The assembled genome sizes of the seven strains showed little variation, ranging from 4.915 to 5.073 Mb, with a consistent GC content between 64.9 and 65.0%. Gene prediction analysis identified between 4,100 and 4,289 genes per strain, including rRNA genes and pseudogenes. These values are comparable to those of previously published *X. euvesicatoria* genomes (Timilsina et al., 2020; Tables 1; Supplementary Table S1). To assess the quality of the genome assemblies, we evaluated metrics such as coverage depth, contiguity, base accuracy, CheckM (Parks et al., 2015), and BUSCO (Manni et al., 2021). The final assembled genomes achieved depths exceeding 200×, ensuring high confidence in assembly accuracy (Supplementary Table S1). CheckM and BUSCO were used to determine genome completeness and contamination. The hybrid approach enhanced assembly contiguity and accuracy, particularly in resolving repetitive regions and plasmid sequences. We identified 1–3 plasmids in each strain, both in the typical and atypical *Xep* strains. The plasmid size varied substantially, ranging from 10 kb to over 255 kb. Notably, all plasmid sequences were completely circularized (Supplementary Table S1), providing a comprehensive view of the plasmid architecture in these strains.

### Low nucleotide sequence divergence of Taiwanese *Xep* strains

**Figure 2:**
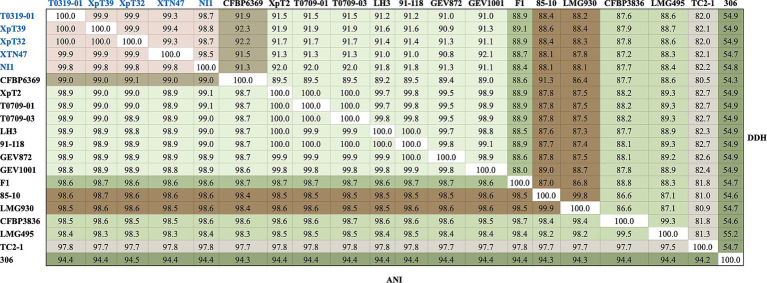
FastANI and dDDH analyses of the selected strains. Strains of the *taiwanensis* lineage are marked in blue, whereas different species are marked in various colors. The complete FastANI and dDDH analyses of 139 strains are shown in Supplementary Figure S1.

To clarify the phylogenetic positions and genetic diversity of the sequenced Taiwanese *Xep* strains, we used multiple complementary approaches to analyze their genome sequences. We compared the average nucleotide identity using FastANI (v.1.2) (Jain et al., 2018) and digital DNA–DNA hybridization (dDDH) (Meier-Kolthoff et al., 2022) of 139 strains, including *Xep* reference strain 91–118 and *Xanthomonas euvesicatoria* pv. *euvesicatoria* (*Xee*) reference strain 85–10 (Supplementary Table S2). The commonly accepted thresholds for species delineation are ANI ≥ 95% and dDDH ≥70% (Chun et al., 2018). The three typical *Xep* strains from Taiwan (XpT2, T0709-01, and T0709-03) and strain 91–118 had ANI scores of 100.0% and dDDH values of 99.8–100.0%, indicating great similarity in their genomes (Figure 2). These findings are consistent with those of previous studies (Barak et al., 2016; Constantin et al., 2016; Bansal et al., 2018; Jibrin et al., 2018; Fan et al., 2022), indicating that *Xep* differs from *Xee* based on an ANI score > 98.5% and a dDDH value >87.3% (Figure 2).

Surprisingly, the four atypical strains sequenced in this study (T0319-01, XpT39, XpT32, and XTN47) and the Nigerian strain NI1 had ANI scores of 99.8–100.0% and dDDH values of 98.6–100.0%, revealing high similarity among their genomes (Figure 2). These four strains were the most closely related to *X. euvesicatoria* pv. *allii* CFBP6369 when compared with other *X. euvesicatoria* strains. The ANI scores between the four strains were 99.0–99.1% and the dDDH values were 91.3–92.3%, supporting the results of the phylogenomic analysis (see Phylogenomics analysis). Additionally, the four strains had ANI scores of 98.5–98.6% and dDDH values of 88.0–88.4% compared to *Xee* strains, and ANI scores of 98.8–99.0% and dDDH values of 90.7–91.8% compared to *Xep* strains (Figure 2).

### Phylogenomics analysis confirmed the phylogenic positions

**Figure 3:**
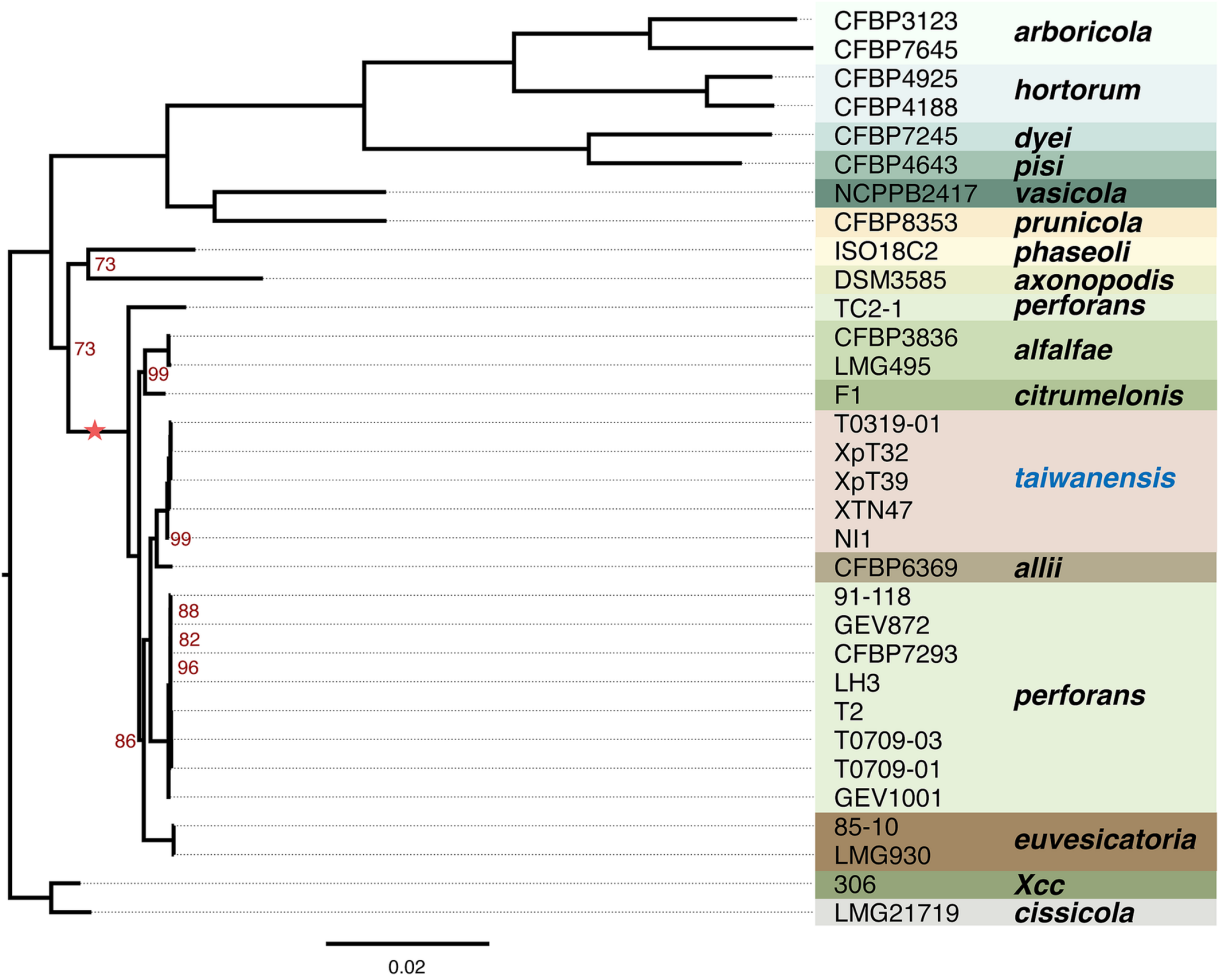
Phylogenetic analysis. The maximum likelihood phylogenetic tree was constructed based on concatenated core genes from 1,346 single-copy genes across 32 genomes rooted in *X. citri* subsp. *citri* strain 306. The scale bar represents substitutions per site. The tree was generated using RAXML-NG with 1,000 bootstrap replicates. Branch support values of less than 100% are indicated by red numbers. The complete phylogenetic analysis of the 139 genomes is provided in Supplementary Figure S2. The asterisk indicates the lineage used in recombination analysis.

To further understand the phylogenetic positions of these Taiwanese *Xep* strains and their relationship with other pathovars of *X. euvesicatoria*, we compared the protein-coding genes across 139 strains. Using the ortholog inference method Markov Cluster Algorithm (MCL) (Enright et al., 2002), 8, 580 gene families were identified. Based on the presence or absence of gene families in each genome, we identified 1,642 core gene families (present in all strains, regardless of copy number) and a pangenome of 6,938 gene families (present in at least one strain) (Figure 3; Supplementary Figure S2). For phylogenetic analysis, 1,346 orthologous genes with a strict one-to-one single-copy relationship were used to construct the phylogenetic tree. In agreement with the nucleotide sequence comparison, the three typical *Xep* strains of this study (XpT2, T0709-01 and T0709-03) formed a well-supported clade with published *Xep* strains, including the reference strain 91–118, and were separated from *Xee*. On the other hand, the four atypical *Xep* strains of this study (T0319-01, XpT39, XpT32, and XTN47) and seven published strains (XVP-272, XVP-270, XVP-273, XVP-271, XVP-267, XVP-265 and Xant-477) (Chen et al., 2024) that were also collected in Taiwan and the atypical *Xep* strain NI1 from Nigeria formed a separate branch on the phylogenetic tree (Figure 3; Supplementary Figure S2). This suggests that, together with the published NI1 genome, these strains form a new lineage of *X. euvesicatoria*. This new lineage is phylogenetically closer to *X. euvesicatoria* pv. *allii* than to *Xep* (Figure 3; Supplementary Figure S2).

**Figure 4:**
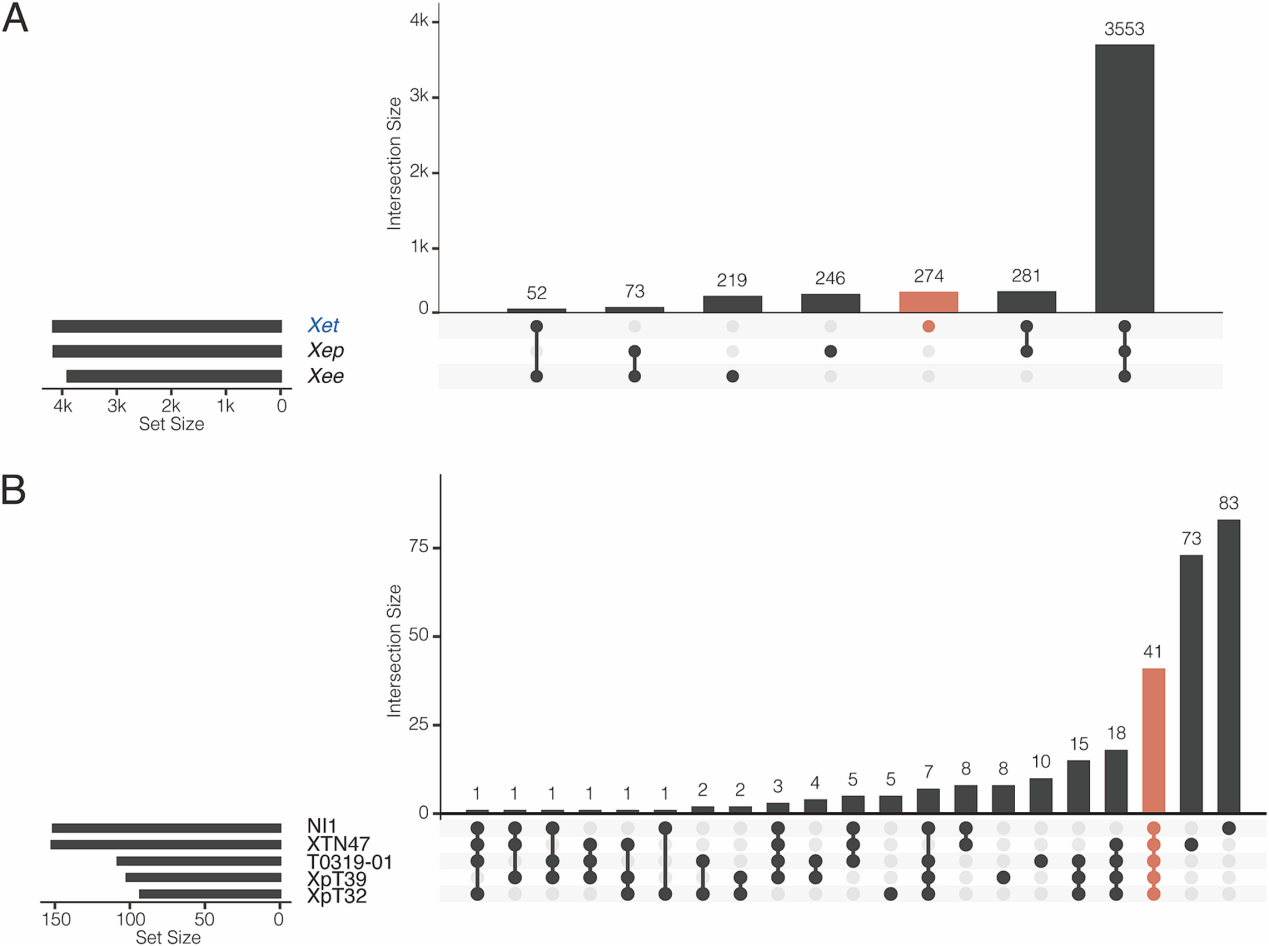
Pangenome analysis of *X. euvesicatoria* lineages. **(A)** Shared gene families among three *X. euvesicatoria* lineage *Xe*e, *Xep* and *Xet.* Left panel: Total number of gene families in each lineage. Right panel: intersection of gene families in each lineage. Each column represents a unique combination of lineages sharing the same gene family, as indicated by the connected dots below the x-axis. The number of gene families in each intersection set is indicated in each column. **(B)** Shared gene families within the *Xet* lineage, including the strains sequenced in this study and the NI1. This plot follows the same principle as in (A).

We further studied the presence and absence of variations in gene families among *Xee*, *Xep*, and the new lineages (Figure 4; Supplementary Figures S3, S4). In total, 3,553 gene families were shared among the three lineages and comprised the core genome (Figure 4A). The new lineage had more unique gene families (274 gene families) than did the other two lineages (219 and 246 in *Xee* and *Xep*, respectively). The number of gene families shared between the new lineage and *Xep* (281) is much higher than those shared between the new lineage and *Xee* (52) or between *Xee* and *Xep* (73). Gene families unique to the new lineage were further analyzed (Figure 4B; Supplementary Figure S4). Of the 274 lineage-specific genes, a limited number were shared between two or more strains, and 41 genes (15%) were shared by five strains. Strains XTN47 and NI1 had 73 and 83 unique genes, respectively, but only 5–8 unique genes were found in each of the other three strains (Figure 4B). The lineage-specific genes in the new *Xep* strains were functionally diverse, with many being involved in key biological processes, such as recombinational repair (GO:0000725), carbohydrate metabolism (GO: 0005975), catalytic activity (GO:0003824), zinc ion homeostasis (GO:0006829), and electron transport chain (GO:0022900). Additionally, these genes contribute to critical cellular functions, such as rRNA processing (GO:0006364), cilium- or flagellum-dependent cell motility (GO:0001539), and response to oxidative stress (GO:0006979), highlighting their potential roles in the adaptation and survival of the new lineage under varying environmental conditions and host interactions (Supplementary Figure S3).

### Homologous recombination of *Xanthomonas euvesicatoria* strains

To infer recombination events, we reconstructed a reference phylogeny using single-copy gene families across *Xep* strains. Recombination was assessed by analyzing concatenated universal genomic regions common to the compared genomes in the whole-genome alignment. ClonalFrameML was employed to estimate the rates of homologous recombination among the three lineages of *X. euvesicatoria*. The analysis revealed that within atypical *Xep* strains, including NI1, the rate of homologous recombination involving imported DNA was less than half the rate of mutation (R/*θ* < 0.5; Table 2). However, when we included other *Xep* strains in the comparison, the recombination rate increased, suggesting more frequent genetic exchange across these groups. Conversely, when atypical *Xep* strains were excluded, the remaining *Xep* strains exhibited a recombination rate 1.5 times higher than their mutation rate (R/θ > 1.5). Notably, the average size of the recombination fragments (*δ*) decreased significantly upon the inclusion of atypical and other typical *Xep* strains. This reduction indicated substantial recombination events between the lineages of atypical *Xep* strains and other typical *Xep* strains, possibly due to horizontal gene transfer. In all scenarios, the effect of recombination on nucleotide variation (measured as R/m) was greater than that of mutations (R/m > 1), underscoring the dominant role of recombination in the genetic diversity of *X. euvesicatoria* populations.

### Phenotypic characteristics of Taiwanese *Xep* strains

Based on the *gapA* phylogeny, *Xep* strains isolated in Taiwan in 1996 formed three distinct groups. In addition to the two typical groups of *Xep* strains, several strains isolated in Taiwan since 1996 are phylogenetically close to the representative atypical strain NI1 from Nigeria (NZ_NISG01000001.1; Timilsina et al., 2015), suggesting a potential origin for atypical *Xep* strains in Taiwan. The atypical *Xep* strains of the new lineage were amylolytic, indicating that they can break down starch into simpler sugars through the action of amylase enzymes. This characteristic is shared by *Xep*, but not in the *Xee* (Supplementary Table S3). Additionally, these strains differed from *Xee* and *Xep* in their carbon utilization patterns (Supplementary Table S3). These phenotypic differences suggest that atypical *Xep* strains may represent a divergent lineage, potentially of Taiwanese origin. Together with the previous analysis, these findings further support the idea that these strains form a new lineage and do not belong to the known pathovars of *X. euvesicatoria*. Considering the recent progress in defining intra-species units (Rodriguez-R et al., 2024; Viver et al., 2024), we propose to consider these atypical strains as *X. euvesicatoria* genomovar *taiwanensis* (*Xet*, See Discussion).

### Plasmid diversity and horizontal gene transfer of heavy metal resistance gene clusters

**Figure 5:**
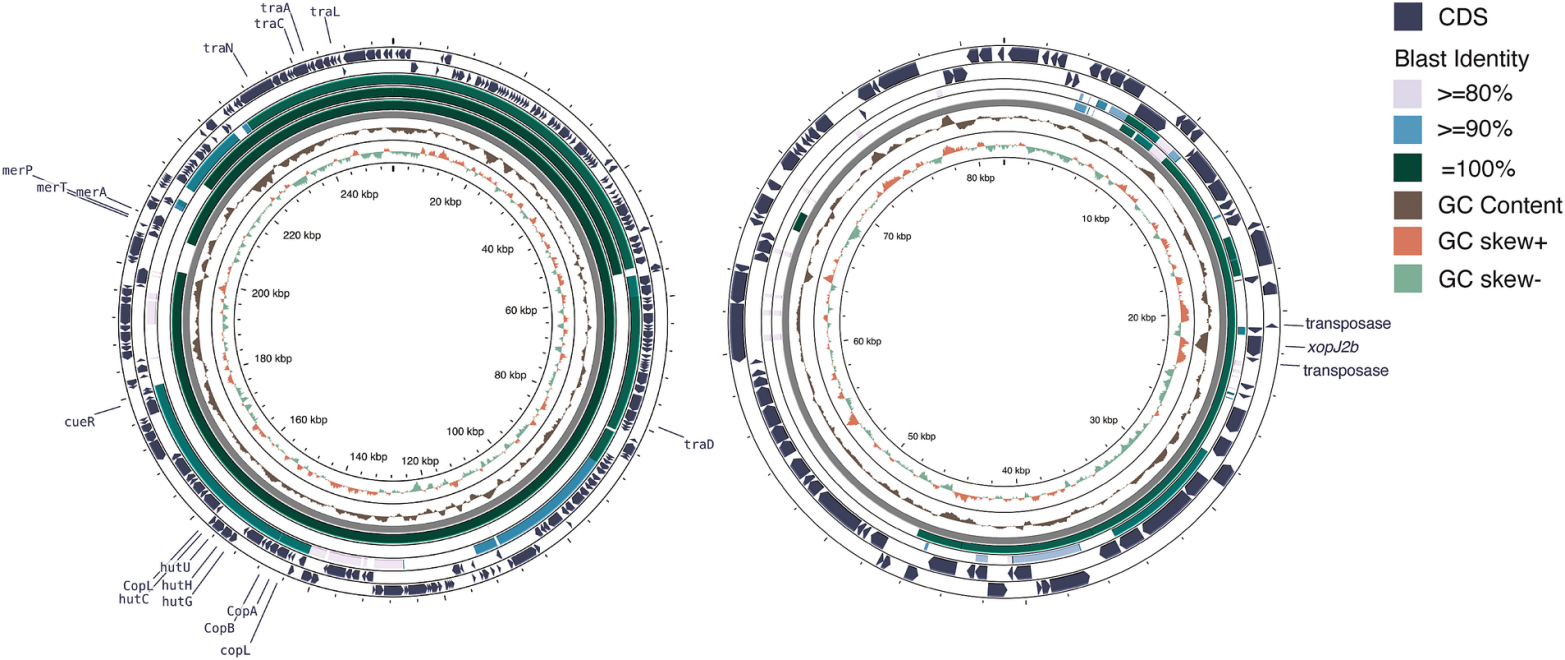
Plasmid comparison analysis. **(A)** Copper resistance-related plasmid comparison. From the outermost to innermost layers: coding sequences (CDS) of pT0319-01.1 on both strands, BLAST comparison results with pT0709-01.1, pXpT2.1, and pLH3.1, followed by GC content, GC skew, and grid layers. The window size for GC content and GC skew analysis was 10 kb with a step size of 10 bp. **(B)** Plasmid profiles illustrating horizontal gene transfer and fusion. From the outermost to the innermost layers: CDS of pT0709-01.2 on both strands, BLAST comparison results with pT0319-01.3 and pXAC64, followed by GC content, GC skew, and grid layers. The window size for GC content and GC skew analysis was 500 bp with a step size of 1 bp. Illustration created using Proksee (Grant et al., 2023).

We identified eight plasmids in three typical *Xep* strains and 10 plasmids in four *Xet* strains (Supplementary Table S1). All strains carried at least one additional plasmid in addition to the Cu^R^ plasmid. In typical *Xep* strains, a consistent 82-kb plasmid was identified, appearing to be a fusion of a 63-kb plasmid from *Xet* strains and a 64-kb plasmid from *X. campestris* pv. *campestris* CFBP6943 (GenBank accession number: CP066920.1) (Dubrow et al., 2022; Table 1). Smaller plasmids of 28 kb and 10.2 kb were also identified, with the 10.2 kb plasmid being a derivative of the 28 kb plasmid (Webster et al., 2020). Notably, the plasmid pXpT2.1, derived from the Cu^R^ plasmid, lacked a 160-kb region carrying heavy metal resistance genes (Figure 5A).

In *Xe*t strains, 63-kb plasmids, similar to pXAC64 of *X. citri* pv. *citri* (GenBank: NC_003922.1) were identified (56–61% coverage and > 95% identity) (da Silva et al., 2002) (Figure 5B). Strain XTN47, isolated in 1996, carried a 97-kb plasmid, pXTN47, but lacked a 63-kb plasmid. Sequence analysis revealed that pXTN47 is a fusion of the 63-kb plasmid and a 34-kb region from the 67-kb plasmid pLMG696-1 of *X. citri* pv. *durantae* LMG696 (GenBank accession number: CP066344.1) (Rana et al., 2022), suggesting a homologous recombination-mediated plasmid fusion. Collectively, either the 63-kb plasmid or its fused derivative was present in all the sequenced strains. Additionally, the 115-kb plasmid pT0319-01.2 was highly similar to the plasmid pF of *X. citri* pv. *phaseoli* var. *fuscans* (GenBank: CP021016.2; 94% coverage, 99.9% identity) and pF of *X. citri* pv. *citri* DAR73910 (GenBank accession number: CP060477.1) (Webster et al., 2020) (Figure 5B). A 30-kb plasmid pXpT39.3 was similar to pXAG27 of *X. citri* pv. *glycines* (GenBank accession number: CP026335.1; 94% coverage and > 96.6% identity) (Carpenter et al., 2019).

The four Cu^R^ strains possessed a large ~250 kb plasmid containing the Cu resistance gene cluster and were absent in the Cu^S^ strains. This megaplasmid showed high nucleotide sequence identity (>99%) with Cu^R^ strains in pT4p2 of *X. citri* subsp. *citri* (Gochez et al., 2018; Huang et al., 2021) and pLH3.1 of *Xep* (Richard et al., 2017) (Figure 5A). Each plasmid contained a complete set of copper resistance gene clusters, including *copLABGF*, but lacked *copCD* (Huang et al., 2021). Additionally, a mercury-resistance operon (*merRTPA*) was flanked by an ISL3-like transposon, suggesting transposition. Unlike pT4p2, these plasmids did not contain an ~40 kb inverted repeat or arsenate resistance gene cluster.

The copper resistance plasmids also contained genes encoding heavy metal resistance ATP-binding cassette (ABC) transporters (*cusABC* and *czcABC* clusters) and a CadR/PbrR-like transcription regulator near a *czcABC* cluster. A complete *Tra* gene cluster encoding 16 Tra proteins essential for plasmid mobility was found in the Cu^R^ plasmids and pXpT2.1. The presence of *Tra* genes with high sequence similarity to other plasmids suggested that heavy metal resistance genes were likely horizontally transferred between xanthomonads.

To further test the mobility of copper resistance genes, they were transferred from Cu^R^ strain T0319-01 to Cu^S^ strain XTN47Rif through bacterial conjugation. The resulting transconjugants showed the same level of copper resistance as donor strain T0319-01. The frequency of conjugative gene transfers ranged from 10^−7^ to 10^−5^ transconjugants per recipient.

We also screened two populations of *X. euvesicatoria* strains collected from Taiwan at different times (Chen et al., 2024; Parajuli et al., 2024). The analysis revealed that the frequency of the copper resistance gene cluster increased in the strains collected in recent years, whereas earlier collections showed a lower frequency (Supplementary Figure S5). This indicates the recent acquisition and spread of copper resistance among *X. euvesicatoria* populations in Taiwan, likely driven by selective pressure from the use of copper-based bactericides.

### Composition of lipopolysaccharides biosynthetic gene clusters

Lipopolysaccharides (LPSs) produced by *Xee* 85–10 are known to trigger basal defense responses in pepper (Keshavarzi et al., 2004). Comparative genomic analysis showed that LPS biosynthetic gene clusters, located between the conserved genes cystathionine gamma-lyase (*metB*) and electron transport flavoprotein subunit A (*etfA*), exhibited high variability among the four lineages of bacterial spot *Xanthomonas.* Specifically, the atypical *Xep* strain NI1 differed significantly from the reference strain 91–118 in both gene content and cluster number (Potnis et al., 2011; Jibrin et al., 2018; Jibrin et al., 2022). Three typical *Xep* strains from Taiwan had LPS biosynthetic gene clusters identical to those of *Xep* 91–118 (Supplementary Figure S6). Additionally, a unique LPS biosynthetic gene cluster in NI1 was consistently present in the *Xet* lineage (Supplementary Figure S6). These findings highlight the distinct composition of LPS biosynthetic gene clusters in the new lineage compared to typical *Xep* strains.

### Type III secretion system and effectors recombination events

In *Xanthomonas*, the type III secretion system (T3SS) is essential for pathogenicity because it modulates host plant physiology and enables evasion of host immune responses (Timilsina et al., 2020). All seven sequenced strains processed conserved T3SS gene clusters encoding *hrp* (hypersensitive response and pathogenicity), *hrc* (*hrp* conserved), and *hpa* (*hrp* associated), indicating that T3SS is highly conserved among these strains.

We examined the allelic diversity of three effector genes, *xopAF*, *xopJ2a*, and *xopJ4*, which determine the tomato races and host ranges in *Xep* strains (Astua-Monge et al., 2000a; Astua-Monge et al., 2000b; Timilsina et al., 2016). The three typical *Xep* strains contained an intact *xopJ4* gene (syn. *AvrXv4*), which specifically determines the tomato race T4 in *Xep* (Astua-Monge et al., 2000b). An early stop codon was found in *xopAF* (syn. *AvrXv3*), the effector responsible for eliciting Xv3 resistance (Astua-Monge et al., 2000a), classifying these *Xep* strains as the tomato race T4 (Jibrin et al., 2022) (Supplementary Figure S7).

**Figure 6:**
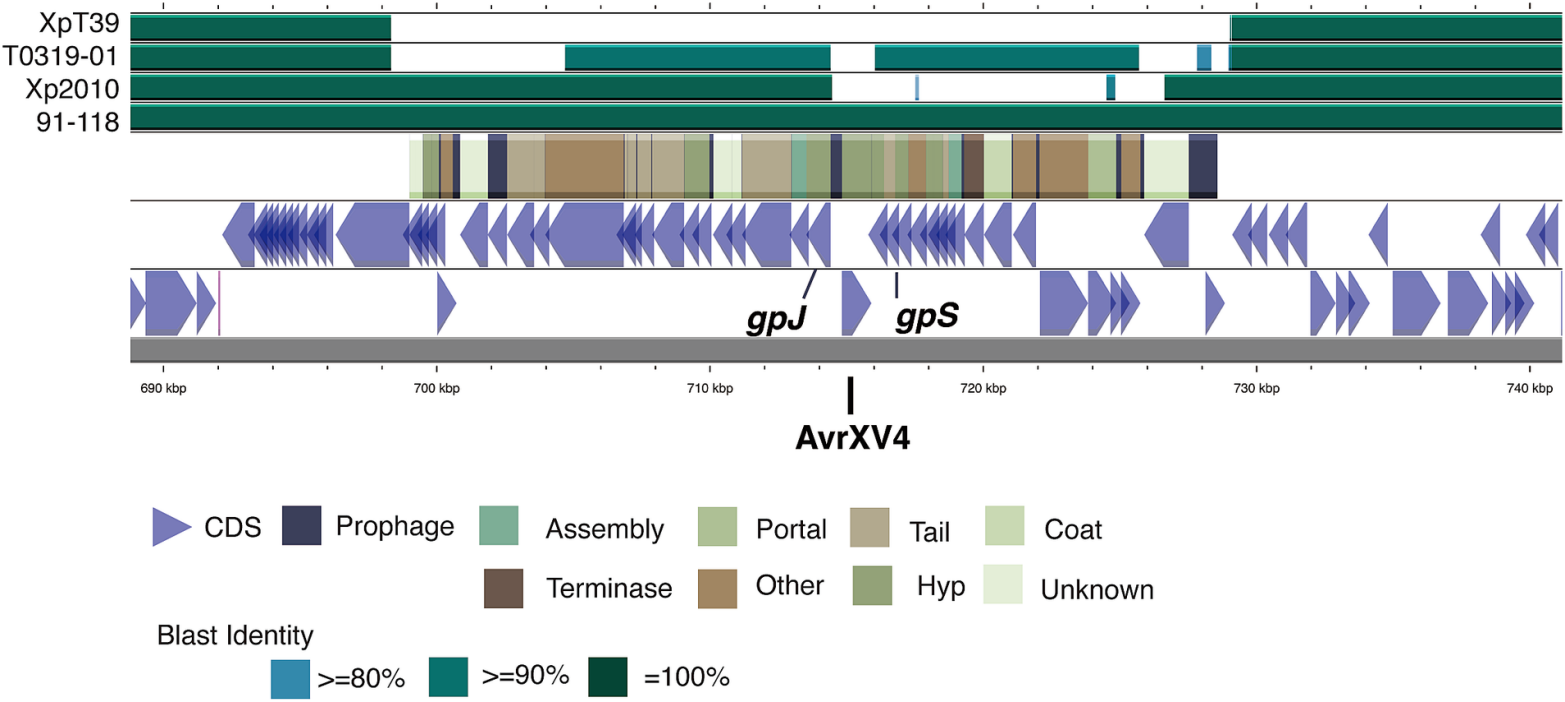
Diversity of the *xopAF* flanking region among the different strains. The diagram illustrates the genomic structure from lower to upper sections, including coding sequences (CDS) on both strands of the T0709-01 chromosome, prophage-related genes, and BLAST comparison results with strain 91–118, Xp2010, T0319-01, and XpT39. The color coding in the BLAST results indicates sequence identity levels, with darker shades representing higher identity. The positions of the *xopAF* gene (syn. *AvrXV3*) is marked within the prophage region, highlighting the variability in this region across different strains. Illustration created using Proksee (Grant et al., 2023).

In contrast, five strains of the new lineage *Xet*, including the Nigerian strain NI1 (Jibrin et al., 2022), possess *xopAF*, but lack *xopJ4*. Within this lineage, two distinct *xopAF* alleles were observed. Strains XTN47 and NI1 have an intact *xopAF* gene, classifying strain XTN47 as tomato race T5, similar to NI1 (Jibrin et al., 2022). However, in the other three strains, the C-terminus of the *xopAF* coding sequence was truncated due to the insertion sequence ISXac4. Additionally, all seven sequenced strains and NI1 contained *xopJ2b*, a homolog of *xopJ2a* with 71% amino acid identity (Iruegas-Bocardo et al., 2018; Potnis, 2021; Jibrin et al., 2022). The *xopJ2b* gene was consistently present in these strains, located on the 63-kb plasmid or its derived plasmids, whereas *xopJ2a* was absent (Figure 6). These findings highlight conserved T3SS gene clusters but significant allelic diversity and host specificities among different *X. euvesicatoria* lineages.

Further analysis showed that the *xopJ4* gene in the three typical *Xep* strains was located within a P2-like temperate prophage region between the *gpJ* and *gpS* genes (Figure 6). This structure, including *xopJ4*, is conserved in typical *Xep* strains and the reference strain 91–118 (Timilsina et al., 2019) but is absent in the *Xet* strains. Interestingly, another P2-like prophage sharing 92% identity with the prophage carrying *xopJ4* was identified in the same chromosomal region of the new lineage strains T0319-01 and XTN47, but no effector genes were found. The plasmid-borne *xopJ2b* gene is consistently flanked by two insertion sequence elements. These findings suggest significant recombination events and horizontal gene transfer involving type III effectors among the different *X. euvesicatoria* lineages.

### Novel arrangement of type IV secretion gene clusters

We identified two copies of the VirB/VirD4 type IV secretion system (T4SS) cluster in typical *Xep* strains from Taiwan. The first one, a complete cluster with 12 genes ending with *virD4*, was located on the chromosome and showed high protein sequence similarity (90–100%) with the T4SS of the *Xep* reference strain 91–118 (Supplementary Table S4). The second cluster, consisting of nine genes lacking *virB1* and *virD4*, was found on the 82-kb plasmids (Supplementary Table S4). These plasmid-borne T4SS genes have low protein sequence similarity with those of reference strains *Xep* 91–118, *Xee* 85–10, and *X. citri* subsp. *citri* 306. Notably, the three typical *Xep* strains lacked the Dot/Icm T4SS cluster (Supplementary Table S5).

In contrast, each strain of the new lineage harbored at least two distinct VirB/VirD4 T4SS clusters (Supplementary Table S4). The first T4SS cluster, a chromosomal cluster of 12 genes ending with *virD4*, shares 90–100% protein sequence similarity with the chromosomal T4SS in *Xep*, indicating strong conservation within the lineage (Supplementary Table S5). This cluster was also present on the NI1 chromosome, further distinguishing this new lineage from typical *Xep* strains. Additionally, a second VirB/VirD4 T4SS cluster, lacking *virD4* and consistsing of 10 genes, was consistently found on the 63-kb plasmids and the 97-kb plasmid pXTN47, which includes the 63-kb plasmid backbone. This plasmid-borne cluster shares high protein sequence similarity with the T4SS genes of *Xee* 85–10 but differs from those in *Xep* 91–118, particularly in the VirB4, VirB5, and VirB6 proteins (Supplementary Table S4).

Interestingly, the plasmid-borne T4SS genes in the new lineage were distinct from those in the *Xep* strains in both gene composition and sequence. Proteins such as VirB1, VirB4, VirB6, VirB9, VirB10, and VirB11 showed greater similarity to those in *X. citri* subsp. *citri* 306 than to those in *Xee* 85–10, suggesting a distinct evolutionary pathway (Supplementary Table S4). The third T4SS cluster, found in the plasmids of strains XTN47 and T0319-01, originated from the pLMG696-1 plasmid of *X. citri* pv. *durantae* LMG696, further emphasizing the diversity of T4SS clusters in these strains. These findings highlight the diversity and complexity of T4SS clusters among different *Xep* strains and the new lineage, suggesting their potential role in pathogenicity and host interactions.

## Discussion

Our study provides crucial insights into the taxonomy and phylogenetic relationships within the bacterial spot *of X. euvesicatoria* pathovars by leveraging high-quality, complete genome strains in Taiwan. The availability of circularized chromosomes and plasmids for three typical and four atypical strains enabled detailed comparative genomic analyses, enhancing our understanding of the bacterial identification, evolution, and pathogenicity of bacterial spot xanthomonads. Completely circularized genomes offer a more comprehensive view of dynamic variations at the inter-strain level (Gochez et al., 2018; Kaur et al., 2019; Huang et al., 2021) than draft genome assemblies or resequencing studies (Zhang et al., 2015; Bansal et al., 2018; Patané et al., 2019; Timilsina et al., 2019; Chen et al., 2024; Parajuli et al., 2024). Additionally, the phylogenetic classification of *Xanthomonas* species can be challenging owing to genetic cohesion within the genus and the absence of high-quality genomic resources for certain lineages. For example, Agarwal et al. (2023) reanalyzed 1,910 diverse *Xanthomonas* genomes, leading to the reclassification of 288 previously misclassified strains.

Our analyses revealed that the atypical strains collected in Taiwan and NI1 formed a distinct genetic lineage within *X. euvesicatoria* separated from the known pathovars *X. euvesicatoria* pv. *euvesicatoria* (*Xee*) and *X. euvesicatoria* pv. *perforans* (*Xep*) (Figure 2). The average nucleotide identity (ANI) scores among these atypical strains were > 99.8%, indicating that they belong to the same intra-species unit. Importantly, the ANI scores between the atypical strains and *Xep* or *Xee* ranged from 98.5 to 98.8%, suggesting significant genomic divergence. In *X. euvesicatoria* phylogenetic analysis, an ANI threshold of >98.5% is commonly used to differentiate *Xep* isolated from *Xee* (Barak et al., 2016; Constantin et al., 2016; Bansal et al., 2018; Jibrin et al., 2018; Fan et al., 2022). This observation aligns with the concept of the ´ANI gap´, which refers to a discontinuity in ANI values that helps define intra-species units (Rodriguez-R et al., 2024; Viver et al., 2024). Strains with ANI values above 99.2–99.8% (midpoint 99.5%) can be considered to belong to the same genomovar within a species (Rodriguez-R et al., 2024; Viver et al., 2024). The ANI gap observed between atypical strains and known pathovars supports the designation of a new genomovar.

In plant pathogenic bacteria, ′pathovar′ traditionally describes strains below the subspecies level that differ in pathogenicity based on symptom type or host range. Our data, along with previous studies, indicate that the two known pathovars and the newly identified phylogenetic lineage within the *X. euvesicatoria* complex share highly similar plant host ranges and disease symptoms, particularly in causing bacterial spot disease in tomatoes and peppers (Timilsina et al., 2020). Given the limited phenotypic differences and host range among these three phylogenetic lineages (Bradbury, 1983; Parker et al., 2019; Timilsina et al., 2020), we propose that the term ′genomovar′ is more appropriate than ′pathovar′ for naming these lineages within *X. euvesicatoria*. We recommend using the term ′genomovar′ to designate the three phylogenetic lineages of *X. euvesicatoria* as *X. euvesicatoria* genomovar *euvesicatoria* (*Xee*), genomovar *perforans* (*Xep*), and genomovar *taiwanensis* (*Xet*) with strain T0319-01 as the representative *Xet*. This approach reflects the genetic distinctions among lineages, while acknowledging their similar pathogenic characteristics.

Our data also suggest that the novel genomovar *taiwanensis* of *X. euvesicatoria* likely originated from Taiwan. The earliest identified strain of *Xet*, XTN47, was isolated in 1996, predating the first reported identification of *Xep* strains in tomatoes in 2010 (Leu et al., 2010). It is plausible that the *Xet* strains were subsequently introduced into Nigeria through the global trade of tomato seeds, leading to their presence in that region.

### Plasmids as the recombination vehicle to faciliate the transfer of virulence/avirulence factors or heavy metal resistance genes

Plasmids in *Xanthomonas* species that cause bacterial leaf spot in tomato and pepper vary widely in size and often carry virulence or heavy metal resistance genes (Minsavage et al., 1990; Thieme et al., 2005; Roach et al., 2019; Huang et al., 2021). Unfortunately, detailed information regarding plasmids from bacterial spot *Xanthomonas*, particularly *Xep*, remains limited (Potnis et al., 2011; Roach et al., 2019; Chen et al., 2024; Parajuli et al., 2024). In this study, we found that the 63-kb plasmid in the *Xet* lineage resembles the pathogenicity plasmid pXAC64 of *X. citri* subsp. *citri* (da Silva et al., 2002). The 82-kb plasmid in three typical *Xep* strains likely originated from a recombination event, leading to plasmid cointegration, a phenomenon also observed in *X. citri* subsp. *citri* strains in Taiwan (Huang et al., 2021).

The *copLABGF* cluster associated with heavy metal resistance and the *Tra* cluster for plasmid mobility are located on an approximately 250-kb megaplasmid. Previous conjugative transfer experiments confirmed the mobility of this plasmid, enabling the horizontal transfer of copper resistance genes between *Xet* strains (Basim et al., 2005; Behlau et al., 2012). Additionally, the presences of the mercury resistance gene cluster (*mer*RTPA) instead of the arsenate resistance gene cluster (Huang et al., 2021), suggests transposition events mediated by insertion elements. These findings indicate significant variation in plasmid distribution among *X. euvesicatoria* strains depending on their geographical origin and host, highlighting the diversity and evolutionary significance of plasmid profiles in *Xanthomonas* (Burlakoti et al., 2018; Lai et al., 2021; Xu et al., 2023).

### Evolution of type III effectors

Recombination and other mechanisms likely contribute to the gain and loss of type III secretion system (T3SS) effector genes in *Xanthomonas*. The *xopJ4* gene is carried by a P2-like temperate prophage in the *Xep* genomes, reflecting lysogenic conversion (Harrison and Brockhurst, 2017), which facilitates the horizontal transfer of virulence factors and enhances the fitness of pathogenic bacteria. This process has been observed in both animal pathogens (Harrison and Brockhurst, 2017) and the phytopathogen *Ralstonia solanacearum* (Askora et al., 2017), suggesting its role in the evolution of *Xep* races.

In contrast, the *xopJ2b* gene is consistently flanked by two insertion sequence elements and is located on a virulence-related plasmid. Insertion sequence-mediated transposition may be associated with the gain and loss of *xopJ2a* or *xopJ2b* in the *Xep* strains (Klein-Gordon et al., 2021). The XopJ family of type III effectors, which includes XopJ1, XopJ2a (syn. AvrBsT), XopJ3 (syn. AvrRxv), and XopJ2b (syn. XopJ6 homolog) has been identified in *Xee* and *Xep* (Minsavage et al., 1990; Ma and Ma, 2016; Iruegas-Bocardo et al., 2018; Osdaghi et al., 2021; Potnis, 2021) and plays a role in enhancing bacterial fitness and determining the host range (Timilsina et al., 2016; Osdaghi et al., 2021; Potnis, 2021). XopJ2a can trigger a hypersensitive response (HR) in pepper, limiting the host range of *Xep* to tomato and enhancing *Xep* fitness in tomato plants (Minsavage et al., 1990; Timilsina et al., 2016; Abrahamian et al., 2018). XopJ2b also induces HR in peppers (Iruegas-Bocardo et al., 2018), although its role is not well understood (Sharma et al., 2024).

Since all strains sequenced in this study carried the 63-kb plasmid or its derivatives housing the *xopJ2b* gene, we propose that these plasmids may contribute to the virulence and fitness of the *Xep* and *Xet* strains. Further research is needed to clarify the distribution and roles of *xopJ2b* and these plasmids in *X. euvesicatoria* genomovars (Sharma et al., 2024).

### Recombination of type IV secretion system

The VirB/VirD4 type IV secretion system (T4SS) clusters are widely distributed among xanthomonads responsible for diseases such as bacterial spot in tomato and pepper, citrus canker, and rice bacterial blight (da Silva et al., 2002; Thieme et al., 2005; Potnis et al., 2011; Kaur et al., 2019; Jibrin et al., 2022). Our characterization of these clusters revealed that the new genomovar *Xet* differed significantly from *Xee* 85–10 and *Xep* 91–118. Protein sequence comparisons suggested that the chromosomal T4SS cluster of *Xet* is a hybrid cluster that combines elements from the T4SS clusters of *Xep* and *X. citri* subsp. *citri*.

Interestingly, the Taiwanese strains studied here exhibit substantial variation in their plasmid-borne VirB/VirD4 T4SS proteins compared to each other and *Xep* 91–118, suggesting that they carry a novel VirB/VirD4 T4SS in their plasmids. Furthermore, the plasmid-borne T4SS cluster in *Xet* may have evolved through recombination between the clusters found in the plasmids of *Xee* and *X. citri* subsp. *citri*. Notably, the hybrid plasmid pXTN47 of strain XTN47 harbors two VirB/VirD4 T4SS clusters: one identical to the T4SS cluster of *Xet* strains and the other identical to that in pLMG696-1 of *X. citri* pv. *durantae* LMG696. These findings suggest that plasmids carrying distinct T4SSs may play a critical role in the evolution and adaptation of plant-pathogenic xanthomonads (Rana et al., 2022).

Additionally, strain T0319-01 was the only strain found to carry the Dot/Icm T4SS cluster within the 112-kb plasmid p0319-01.2, which shows high similarity to the plasmid pF of *X. citri* but differs markedly from pXCV182 of *Xee* 85–10. This suggested that *X. euvesicatoria* strains may acquire plasmids carrying the Dot/Icm T4SS cluster from various donors via horizontal gene transfer or other unknown mechanisms (Drehkopf et al., 2023).

## Conclusion

In this study, we performed complete genome sequencing of seven strains of *X. euvesicatoria* genomovars *perforans* (*Xep*) and *taiwanensis* (*Xet*) and the compared them to multiple *Xanthomonas* genomes. This provides evidence of significant diversity and plasticity in both chromosomes and plasmids. These results suggest that the genomovar *taiwanensis* may have originated in Taiwan and subsequently spread to Nigeria through global seed trades. Genomic analysis revealed genetic recombination, evolution of virulence and avirulence factors, and horizontal gene transfer events within the strains of these two genomovars. The gap-free genomes revealed associations between the evolution of type III and type IV effectors and horizontal gene transfer. They also highlighted the role of plasmid diversity and recombination in the genetic landscape of the sequenced strains. Notably, we identified the *cop* gene cluster and mercury resistance gene cluster, but not the arsenate resistance gene cluster, on the megaplasmid of the Cu^R^ strain of *X. euvesicatoria*. Collectively, these findings suggest that horizontal gene transfer and genetic recombination have significantly shaped the genetic makeup of *X. euvesicatoria* genomovars, contributing to their adaptation and survival within the agroecosystem.

## Data Availability

All sequencing data and genome assemblies were deposited in the NCBI database under BioProject ID PRJNA1029321. The *X. euvesicatoria* strains that support the findings of this study are available upon request from CJH. T0319-01^T^ is available in Bioresource Collection and Research Center under ID BCRC 81416 (https://catalog.bcrc.firdi.org.tw/BcrcContent?bid=81416&rowid=1).

## Footnotes

^1^EPPO Global database, 2023 https://gd.eppo.int/taxon/XANTPF/datasheet

^2^ https://github.com/rambaut/figtree/releases
